# Supplementary material for: A 4-year longitudinal study investigating the relationship between flexible school starts and grades
Source: Sci Rep. 2022 Feb 24;12:3178. doi: 10.1038/s41598-022-06804-5 (PMC8873390; doi:10.1038/s41598-022-06804-5)
Supplement: Supplementary file 1 — Supplementary Information. [file 41598_2022_6804_MOESM1_ESM.pdf]

Supplementary information for

A 4-year longitudinal study investigating the relationship between flexible school starts and grades

Authors:

Anna M. Biller[1,2+*], Carmen Molenda[1], Fabian Obster[2§], Giulia Zerbini[1,4], Christian Förtsch[5], Till Roenneberg[1#] & Eva C. Winnebeck[1$*]

*corresponding authors

Affiliations:

[1] Institute of Medical Psychology, Ludwig Maximilian University Munich, Munich, Germany

[2] Graduate School of Systemic Neurosciences, LMU Munich, Germany

[3] Department of Statistics, Statistical Consulting Unit, Ludwig Maximilian University Munich, Munich, Germany

[4] Department of Medical Psychology and Sociology, University of Augsburg, Augsburg, Germany

[5] Biology Education, Faculty of Biology, Ludwig Maximilian University Munich, Munich, Germany

+ current address: Institute of Psychology, Bundeswehr University Munich, Munich, Germany

§ current address: Department of Business Administration, Bundeswehr University Munich, Munich, Germany

# current address: Institute and Polyclinic for Occupational-, Social- and Environmental Medicine, Ludwig Maximilian University Munich, Munich Germany

$ current address: Chair of Neurogenetics, School of Medicine, Technical University of Munich, and Institute of Neurogenomics, Helmholtz Center Munich, Munich, Germany

Corresponding authors:

eva.winnebeck@helmholtz-muenchen.de

anna.biller@med.uni-muenchen.de

This PDF file includes:

Supplementary Figure S1
Supplementary Tables S1-S4

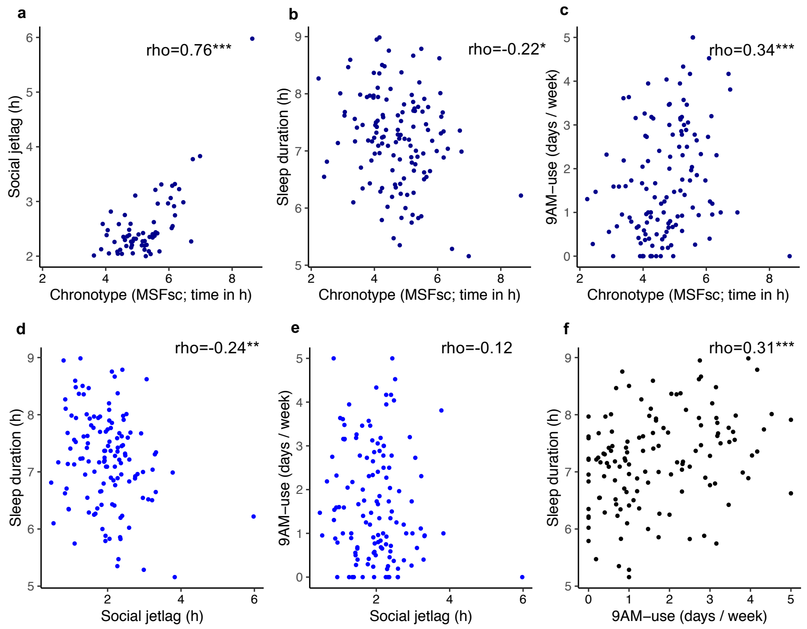

Supplementary Figure S1. Correlations between sleep variables. Spearman rank correlations between the sleep variables social jetlag, chronotype (MSF_{sc}), and sleep duration on schooldays, as well as 9AM-use (frequency of ≥9AM-starts) (n=129). *, p<0.05; **, p<0.01; ***, p<0.001

Supplementary Table S1. Linear mixed regression models 1 and 2: General and system effects on grades. Predicted outcomes are quarterly grades (0%-100%) in 12 academic subjects from students of cohort 1 and 2 (n=157). Abbreviations: b, unstandardized coefficient; se, standard error; beta, standardized coefficient; t, t-statistic; p, p-value; σ^2, variance of residuals of random effects; τ_{00}, variance of ID intercepts of random effects; ICC, intra-class correlation coefficient (describes how much variance is explained by the random effects); N, number of participants; Marginal R^2 describes the amount of variance explained by the fixed effects (predictors); Conditional R^2 describes the amount of variance explained by the full model.

	Model 1					Model 2				
**Predictors**	b	se	beta	t	p	b	se	beta	t	p
(Intercept)	54.11	1.21		44.74	**<0.001**	52.79	1.21		43.56	**<0.001**
System: Flexible system[a]	-0.10	0.42	-0.00	-0.23	0.815	0.64	0.55	0.02	1.16	0.244
Gender: Male[b]	**-4.72**	2.13	-0.12	-2.21	**0.028**	-1.43	2.12	-0.03	-0.67	0.501
Grade level: 7[c]	1.17	0.74	0.02	1.60	0.111	1.23	0.74	0.01	1.66	0.097
Grade level: 8[c]	**3.11**	0.49	0.05	6.39	**<0.001**	**3.15**	0.49	0.05	6.44	**<0.001**
Grade level: 9[c]	**2.59**	0.34	0.06	7.53	**<0.001**	**2.62**	0.35	0.06	7.58	**<0.001**
Grade level: 11[c]	0.50	0.35	0.01	1.42	0.155	0.48	0.36	0.01	1.34	0.180
Grade level: 12[c]	**3.44**	0.55	0.05	6.21	**<0.001**	**3.37**	0.56	0.05	6.05	**<0.001**
Quarter: 2[d]	**0.82**	0.32	0.02	2.59	**0.010**	**0.82**	0.32	0.02	2.58	**0.010**
Quarter: 3[d]	0.30	0.34	0.01	0.88	0.378	0.25	0.34	0.01	0.75	0.451
Quarter: 4[d]	**2.34**	0.33	0.05	7.15	**<0.001**	**2.30**	0.33	0.05	7.00	**<0.001**
Discipline: Sciences[e]	**3.40**	0.30	0.09	11.36	**<0.001**	**5.22**	0.30	0.14	17.30	**<0.001**
Discipline: Social Sciences[e]	**3.87**	0.36	0.08	10.69	**<0.001**	**7.25**	0.37	0.16	19.33	**<0.001**
Male*Sciences	**4.74**	0.54	0.08	8.83	**<0.001**					
Male*Social Sciences	**8.03**	0.65	0.11	12.44	**<0.001**					
Flexible system*Sciences						**-1.23**	0.54	-0.02	-2.29	**0.022**
Flexible system*Social Sciences						**-2.56**	0.63	-0.04	-4.05	**<0.001**
Flexible system*Male						**1.32**	0.52	0.02	2.53	**0.011**
**Random Effects**										
σ^2	200.29					202.07				
τ_{00}	146.38 $_{ID}$					146.37 $_{ID}$				
ICC	0.42					0.42				
N	157 $_{ID}$					157 $_{ID}$				
Observations	16724					16724				
Marginal R^2 / Conditional R^2	0.033 / 0.441					0.028 / 0.436				

[a]Reference is conventional system.
[b]Reference is female.
[c]Reference is grade level 10.
[d]Reference is quarter 1.
[e]Reference is Languages.

Supplementary Table S2. Post hoc results of mixed models 1 and 2. Results are presented as marginal estimated means of quarterly grades scaled 0-100% (standard error), degrees of freedom. Simple contrast results are presented as estimated difference of academic grades (standard error), p-value. Degrees of freedom method: Kenward-Rogers. Results are averaged over the levels of system or gender, grade level, and quarter. Tukey method for comparison of 3 estimates.

Model 1

Gender	Languages	Sciences	Social Sciences	Simple contrasts
**female**	**56.7** (1.19)<br>166	**60.1** (1.19)<br>166	**60.6** (1.21)<br>176	Languages-Sciences:<br>**-3.41** (0.30), **p<.0001**<br>Languages-Social Sciences:<br>**-3.87** (0.36), **p<.0001**<br>Sciences-Social Sciences:<br>**-0.47** (0.36), p=0.3895
**male**	**52.0** (1.79)<br>164	**60.2** (1.78)<br>163	**63.9** (1.80)<br>172	Languages-Sciences:<br>**-8.15** (0.45), **p<.0001**<br>Languages-Social Sciences:<br>**-11.90** (0.54), **p<.0001**<br>Sciences-Social Sciences:<br>**-3.76** (0.52), **p<.0001**
**Simple contrasts**	**4.72** (2.13)<br>p=0.0284	-**0.00** (2.13)<br>p=0.9915	-**3.31** (2.16)<br>p=0.1269	

Model 2

System	Languages	Sciences	Social Sciences	Simple contrasts
**conventional**	54.7 (1.08)<br>168	59.9 (1.08)<br>167	62.0 (1.10)<br>182	Languages-Sciences:<br>**-5.22** (0.30), **p<0.0001**<br>Languages-Social Sciences:<br>**-7.25** (0.38), **p<0.0001**<br>Sciences-Social Sciences:<br>**-2.03** (0.37), **p<0.0001**
**flexible**	56.0 (1.14)<br>212	60.0 (1.13)<br>206	60.7 (1.16)<br>227	Languages-Sciences:<br>**-3.99** (0.45), **p<.0001**<br>Languages-Social Sciences:<br>**-4.69** (0.51), **p<.0001**<br>Sciences-Social Sciences:<br>**-0.69** (0.49), p=0.3355
**Simple contrasts**	**-1.30** (0.54)<br>**p=0.0168**	-0.07 (0.61)<br>p=0.8849	**1.26** (0.61)<br>**p=0.0384**	

System	Female	Male		Simple contrasts
**conventional**	**59.6** (1.18)<br>160	**58.2** (1.77)<br>158		female-male:<br>1.43 (2.12), p=0.5010
**flexible**	**59.0** (1.22)<br>186	**58.9** (1.81)<br>173		female-male:<br>0.11 (2.14), p=0.9580
**Simple contrasts**	0.62 (0.45)<br>p=0.1726	0.7 (0.56)<br>p=0.2106		

Supplementary Table S3. Linear mixed regression models 3a-d: Effect of changes in sleep and of ≥9AM-use on grade improvements from the conventional to the flexible system. Predicted outcomes are quarterly grades (0%-100%) in 12 academic subjects from students of cohort 1 (n=63) over 4 years. “Change” refers to the absolute difference of the respective sleep variable between the conventional and the flexible system (t1-t0). Positive numbers mean later chronotype, longer sleep and more social jetlag in the flexible system (t1). 9AM-use is the frequency of ≥9AM-starts at t1 (no baseline data for calculation of change available). Abbreviations: Flex, Flexible system; b, unstandardized coefficient; se, standard error; beta, standardized coefficient; p, p value; σ^2, variance of residuals of random effects; τ_{00}, variance of ID intercepts of random effects; ICC, intra-class correlation coefficient (describes how much variance is explained by the random effects); N, number of participants; Marginal R^2 describes the amount of variance explained by the fixed effects (predictors); Conditional R^2 describes the amount of variance explained by the full model.

	Model3a: Chronotype change				Model3b: Sleep duration change				Model3c: Social jetlag change				Model3d: 9AM-use			
**Predictors**	b	se	beta	p	b	se	beta	p	b	se	beta	p	b	se	beta	p
(Intercept)	53.26	1.97		**<0.001**	53.30	1.94		**<0.001**	53.29	1.95		**<0.001**	53.35	1.86		**<0.001**
System: Flexible system	-0.01	0.68	0.00	0.983	0.00	0.68	0.00	0.998	0.04	0.68	0.00	0.953	0.02	0.68	0.00	0.977
Gender: Male[a]	-2.56	3.24	-0.07	0.432	-2.64	3.11	-0.07	0.399	-2.64	3.15	-0.07	0.405	-2.80	2.98	-0.07	0.352
Grade level: 7[b]	5.96	3.33	0.02	0.074	6.31	3.35	0.02	0.060	6.46	3.34	0.02	0.053	5.89	3.33	0.02	0.077
Grade level: 8[b]	2.52	0.77	**0.04**	**0.001**	2.49	0.77	**0.04**	**0.001**	2.49	0.77	**0.04**	**0.001**	2.50	0.76	**0.04**	**0.001**
Grade level: 9[b]	2.14	0.57	**0.05**	**<0.001**	2.13	0.57	**0.05**	**<0.001**	2.11	0.57	**0.05**	**<0.001**	2.08	0.57	**0.05**	**<0.001**
Grade level: 11[b]	1.07	0.53	**0.03**	**0.045**	1.08	0.53	**0.03**	**0.042**	1.11	0.53	**0.03**	**0.039**	1.11	0.53	**0.03**	**0.038**
Grade level: 12[b]	3.42	0.82	**0.06**	**<0.001**	3.38	0.82	**0.06**	**<0.001**	3.37	0.82	**0.06**	**<0.001**	3.46	0.82	**0.06**	**<0.001**
Quarter: 2[c]	0.71	0.50	0.02	0.157	0.70	0.50	0.02	0.160	0.71	0.50	0.02	0.155	0.71	0.50	0.02	0.154
Quarter: 3[c]	0.47	0.54	0.01	0.386	0.46	0.54	0.01	0.392	0.47	0.54	0.01	0.380	0.48	0.54	0.01	0.367
Quarter: 4[c]	2.31	0.52	**0.05**	**<0.001**	2.30	0.52	**0.05**	**<0.001**	2.30	0.52	**0.05**	**<0.001**	2.31	0.52	**0.05**	**<0.001**
Discipline: Sciences[d]	6.22	0.40	**0.16**	**<0.001**	6.22	0.40	**0.16**	**<0.001**	6.22	0.40	**0.16**	**<0.001**	6.22	0.40	**0.16**	**<0.001**
Discipline: Social Sciences[d]	7.63	0.48	**0.17**	**<0.001**	7.63	0.48	**0.17**	**<0.001**	7.64	0.48	**0.17**	**<0.001**	7.64	0.48	**0.17**	**<0.001**
Chronotype change (MSF_{sc}; h)	0.40	2.18	0.02	0.855												
Flex* Chronotype change	0.10	0.53	0.00	0.845												
Sleep duration change (h)					2.32	2.94	0.06	0.434								
Flex* Sleep duration change					-0.77	0.83	-0.01	0.352								
Social jetlag change (h)									0.11	2.11	0.00	0.958				
Flex* Social jetlag change									1.28	0.58	**0.03**	**0.027**				
9AM-use (schooldays/week)													-3.04	1.21	**-0.19**	**0.015**
Flex*9AM-use													0.59	0.36	0.02	0.101
**Random effects**																
σ^2	205.07				205.05				204.92				204.99			
τ_{00}	140.27 $_{ID}$				139.10 $_{ID}$				140.24 $_{ID}$				128.08 $_{ID}$			
ICC	0.41				0.40				0.41				0.28			
N	63 $_{ID}$				63 $_{ID}$				63 $_{ID}$				63 $_{ID}$			
Observations	6683				6683				6683				6683			
Marginal R^2 / Conditional R^2	0.040 / 0.430				0.043 / 0.430				0.041 / 0.430				0.071 / 0.428			

[a]Reference is female.
[b]Reference is grade level 10.
[c]Reference is quarter 1.
[d]Reference is Languages.

Supplementary Table S4. Linear mixed regression models 4a-e: Effect of absolute sleep characteristics in the flexible system on grades. Predicted outcomes are quarterly grades (0%-100%) in 12 academic subjects from students of cohorts 1 and 2 (n=129) over 1.5 years in the flexible system. 9AM-use is the frequency of ≥9AM-starts in the flexible system. Abbreviations: b, unstandardized coefficient; se, standard error; beta, standardized coefficient; p, p value; σ^2, variance of residuals of random effects; τ_{00}, variance of ID intercepts of random effects; ICC, intra-class correlation coefficient (describes how much variance is explained by the random effects); N, number of participants; Marginal R^2 describes the amount of variance explained by the fixed effects (predictors); Conditional R^2 describes the amount of variance explained by the full model.

	**Model 4a: Chronotype**				**Model 4b: Sleep duration**				**Model 4c: Social jetlag**				**Model 4d: 9AM-use**				**Model 4e: All**			
**Predictors**	b	se	beta	p	b	se	beta	p	b	se	beta	p	b	se	beta	p	b	se	beta	p
(Intercept)	59.50	5.81		**<0.001**	65.13	10.70		**<0.001**	53.68	3.40		**<0.001**	60.50	2.19		**<0.001**	64.77	14.51		**<0.001**
Gender: Male[a]	-0.31	2.61	-0.01	0.907	-0.61	2.54	-0.01	0.810	-0.83	2.54	-0.02	0.744	-0.72	2.49	-0.02	0.773	-0.15	2.63	0.00	0.956
Grade level: 9[b]	3.35	0.82	**0.05**	**<0.001**	3.35	0.82	**0.05**	**<0.001**	3.36	0.82	**0.05**	**<0.001**	3.27	0.82	**0.05**	**<0.001**	3.27	0.82	**0.05**	**<0.001**
Grade level: 11[b]	0.47	0.62	0.01	0.450	0.46	0.62	0.01	0.451	0.45	0.62	0.01	0.469	0.58	0.62	0.01	0.350	0.57	0.62	0.01	0.355
Grade level: 12[b]	0.02	1.00	0.00	0.984	0.02	0.99	0.00	0.984	-0.02	0.99	0.00	0.983	0.27	1.00	0.01	0.790	0.26	1.00	0.01	0.794
Quarter: 2[c]	0.38	0.65	0.01	0.559	0.38	0.65	0.01	0.559	0.38	0.65	0.01	0.559	0.38	0.65	0.01	0.562	0.38	0.65	0.01	0.561
Quarter: 3[c]	-0.05	0.65	-0.00	0.933	-0.06	0.65	-0.00	0.932	-0.07	0.65	0.00	0.917	0.02	0.65	0.00	0.980	0.02	0.65	0.00	0.979
Quarter: 4[c]	1.69	0.61	**0.04**	**0.005**	1.69	0.61	**0.04**	**0.005**	1.68	0.61	**0.04**	**0.006**	1.76	0.61	**0.05**	**0.004**	1.76	0.61	**0.05**	**0.004**
Discipline: Sciences[d]	4.27	0.43	**0.11**	**<0.001**	4.28	0.43	**0.11**	**<0.001**	4.27	0.43	**0.11**	**<0.001**	4.27	0.43	**0.11**	**<0.001**	4.28	0.43	**0.11**	**<0.001**
Discipline: Social Sciences[d]	4.65	0.49	**0.10**	**<0.001**	4.65	0.49	**0.10**	**<0.001**	4.65	0.49	**0.10**	**<0.001**	4.65	0.49	**0.10**	**<0.001**	4.65	0.49	**0.10**	**<0.001**
Chronotype (local time in h)	-0.53	1.23	-0.03	0.665													-2.37	2.35	-0.12	0.315
Sleep duration (h)					-1.12	1.47	-0.05	0.448									-0.27	1.61	-0.01	0.865
Social jetlag (h)									1.76	1.54	0.07	0.256					3.70	2.78	0.14	0.186
≥9AM-use (schooldays/week)													-2.12	0.91		**0.022**	-1.32	1.20	-0.08	0.272
**Random Effects**																				
σ^2	168.88				168.87				168.87				168.87				168.87			
τ_{00}	174.27 $_{ID}$				173.89 $_{ID}$				172.93 $_{ID}$				167.17 $_{ID}$				168.81 $_{ID}$			
ICC	0.51				0.51				0.51				0.50				0.50			
N	129 $_{ID}$				129 $_{ID}$				129 $_{ID}$				129 $_{ID}$				129 $_{ID}$			
Observations	5111				5111				5111				5111				5111			
Marginal R^2 / Conditional R^2	0.018 / 0.517				0.019 / 0.517				0.022 / 0.517				0.037 / 0.516				0.042 / 0.521			

[a]Reference is female.
[b]Reference is grade level 10.
[c]Reference is quarter 1.
[d]Reference is Languages.
